# 

**Published:** 1850-11

**Authors:** 


### CONTENTS

OF NO. V.

---

### ORIGINAL COMMUNICATIONS.

ESSAYS AND CASES.

ART I.—A Report on Typhoid Fever, made to the Scott County Medical Society. By W. L. SUTTON, M. D , of Georgetown, Ky. - - - - - - - 369

ART. II.—Case of Excision of the Uterus. By PAUL F. EVE, M. D., Prof. of Surg. in Med. College of Georgia. With Remarks, by C. D. MEIGS, M. D., Prof. of Obstetrics and Diseases of Women and Children in Jeff. Med. Coll. of Philadelphia, - - - - - - 401

REVIEWS.

ART. III.—A Systematic Treatise, Historical, Etiological, and Practical, on the Principal Diseases of the Interior Valley of North America, as they appear in the Caucasian, African, Indian, and Esquimaux varieties of its Population. By. DANIEL DRAKE, M. D. - - - 408

SELECTIONS FROM AMERICAN AND FOREIGN JOURNALS.

Cases of Asthma successfully treated by Nitric Acid, - 439

Chloroform in Puerperal Convulsions, - - - - - 441

Laryngotomy and Tracheotomy, - - - - - 442

Case in which the Urachus remained open, and a ring-shaped Calculus, formed on a curved hair in the Bladder, was extracted through the Umbilicus, - - - -443

Observations on the Emmenagogue properties of Polygala Senega, 445
Treatment of Aneurism by Compression, - - - - 448
Case of Uterine Pregnancy of over twenty years standing, - 449
Power of Lupulin in allaying Irritation of the Genital Organs, 451
External Employment of Liquor Chlorini, - - - - 452

ORIGINAL INTELLIGENCE.

Transylvania Medical School, - - - - - - 453
Foreign Correspondence, - - - - - - - 455
Use and Abuse of the Speculum, - - - - - 457
Prof. Paul F. Eve's Clinical Surgery, - - - - 457
Proceedings of the American Medical Association, - - 457
Puffing of Medical Professors, and the arts of Quackery, - 458
Chronic Diarrhea, - - - - - - - - 460
Surgical Instruments. - - - - - - - 460

